# Selective autophagy, lipophagy and mitophagy, in the Harderian gland along the oestrous cycle: a potential retrieval effect of melatonin

**DOI:** 10.1038/s41598-019-54743-5

**Published:** 2019-12-09

**Authors:** Marina García-Macia, Adrián Santos-Ledo, Beatriz Caballero, Adrian Rubio-González, Beatriz de Luxán-Delgado, Yaiza Potes, Susana Mª. Rodríguez-González, José Antonio Boga, Ana Coto-Montes

**Affiliations:** 1 Institute of Cellular Medicine, Newcastle University, William Leech Building, NE2 4HH Newcastle Upon Tyne, UK; 2 Institute of Genetic Medicine, Newcastle University, International Centre for Life Central Parkway, NE1 3BZ Newcastle Upon Tyne, UK; 3 Departamento de Morfología y Biología Celular, Área de Biología Celular, Facultad de Medicina, Universidad de Oviedo, Julián Clavería s/n, 33006 Oviedo, Spain; 4 Servicio de Microbiología, Hospital Universitario Central de Asturias, Avenida de Roma s/n., 33011 Oviedo, Spain; 5 Present Address: Barts Cancer Institute-Queen Mary, University of London, Centre for Tumour biology, John Vane Science Centre, Charterhouse Square, London, EC1M 6BQ UK; 6 Present Address: Instituto de Investigación Biomédica de Salamanca (IBSAL), Neuroenergetics and Metabolism Group, Institute of Functional Biology and Genomics, University of Salamanca-CSIC, Zacarias Gonzalez, 2, 37007 Salamanca, Spain

**Keywords:** Macroautophagy, Mitophagy, Reproductive biology, Lysosomes

## Abstract

Sexual dimorphism has been reported in many processes. However, sexual bias in favour of the use of males is very present in science. One of the main reasons is that the impact of hormones in diverse pathways and processes such as autophagy have not been properly addressed *in vivo*. The Harderian gland is a perfect model to study autophagic modulation as it exhibits important changes during the oestrous cycle. The aim of this study is to identify the main processes behind Harderian gland differences under oestrous cycle and their modulator. In the present study we show that redox-sensitive transcription factors have an essential role: NF-κB may activate SQSTM1/p62 in oestrus, promoting selective types of autophagy: mitophagy and lipophagy. Nrf2 activation in dioestrus, leads the retrieval phase and restoration of mitochondrial homeostasis. Melatonin’s receptors show higher expression in dioestrus, leading to decreases in pro-inflammatory mediators and enhanced Nrf2 expression. Consequently, autophagy is blocked, and porphyrin release is reduced. All these results point to melatonin as one of the main modulators of the changes in autophagy during the oestrous cycle.

## Introduction

Sex bias is still a clear issue in biomedical research, although sexual dimorphism has been described in many cellular processes under pathological and physiological conditions^1,2^. Sex-dependent differences in the activation of autophagy have been reported mainly *in vitro*^3,4^. It has been reported that hormonal changes stimulate autophagy in mammary gland, but just using *in vitro* models^5,6^. Androgens also modulate autophagy in flank organ, which is a sebaceous gland, *in vivo*^7^. However, how autophagy is modulated under sexual hormone variations, as during the oestrous cycle, is poorly understood.

The Syrian hamster Harderian gland (HG) is a tubule-alveolar orbital gland formed by two main types of cells (type I and II) that secretes lipids favouring lubrication of the cornea^8^. The HG has other functions including the production of pheromones^9^, functions related to the pineal-gonadal axis^10^, and synthesis of indolamines^11^. HG exhibits marked sexual differences in relation to cell type and porphyrin production. In previous studies, we have demonstrated alterations of female gland activity during the oestrous cycle^12^. Interestingly, the oestrus phase presents the highest porphyrin–production activity, which has been associated with a prominent oxidative damage to proteins, together with a lower total antioxidant activity and higher autophagy, compared to the dioestrus phase^4,12^. By contrast, the dioestrus phase shows a lower porphyrins production causing reduced oxidative stress levels, a decrease in proteolytic activities and a blockage of autophagy due to activation of mTOR^12^. Morphological changes also occur during the oestrous cycle, the Type II cells of the Harderian gland are more abundant during oestrus^12^.

The main difference between Type I and II cells is the size of the lipid droplets (LD). Type II cells present bigger and more abundant LDs^13^, suggesting that the LDs are active organelles in the HG. Previously, it has been reported that autophagy controls the mobilization of LDs to lysosomes^14,15^. This new type of LD degradation is called lipophagy^14,15^. Although it was first discovered in liver, it has also been observed in several cell types, where it acts as an energy source with the peculiar capability to provide large amounts of free fatty acids in a short time^14,15^. Turnover of endogenous lipid stores is essentially developed by Lysosomal acid lipase (LAL)^16,17^, which could be considered an essential lipophagy marker. However, the presence of lipophagy in HG is still unconfirmed, even though lipid droplets are indispensable components of glandular cells.

The pineal melatonin is the endogenous synchronizer of the circadian rhythms in organisms, mainly of those related to the control of seasonal reproductive phenomena^18^. Likewise, melatonin and its metabolites are well-known antioxidants^19^. Moreover, melatonin is able to maintain mitochondrial homeostasis^20,21^, as it increases the activity of the mitochondrial respiratory complexes I and IV, which result in increased ATP production^22^. Melatonin plays and important role modulating the morphology and physiology of the HG^11,23^. Melatonin secretion depends on the phase of the oestrous cycle^24^ and it is diminished by gonadal steroid hormones^25^.

Based on these findings and considering the observed oscillations in autophagy activity as well as Type II cell abundance along the oestrous cycle^12^, the aim of this study was to identify the main modulators behind autophagic changes in the oestrous cycle and the involvement of the lipophagy.

## Results

### Melatonin receptors expression: cellular response to oxidative stress

**Figure 1:**
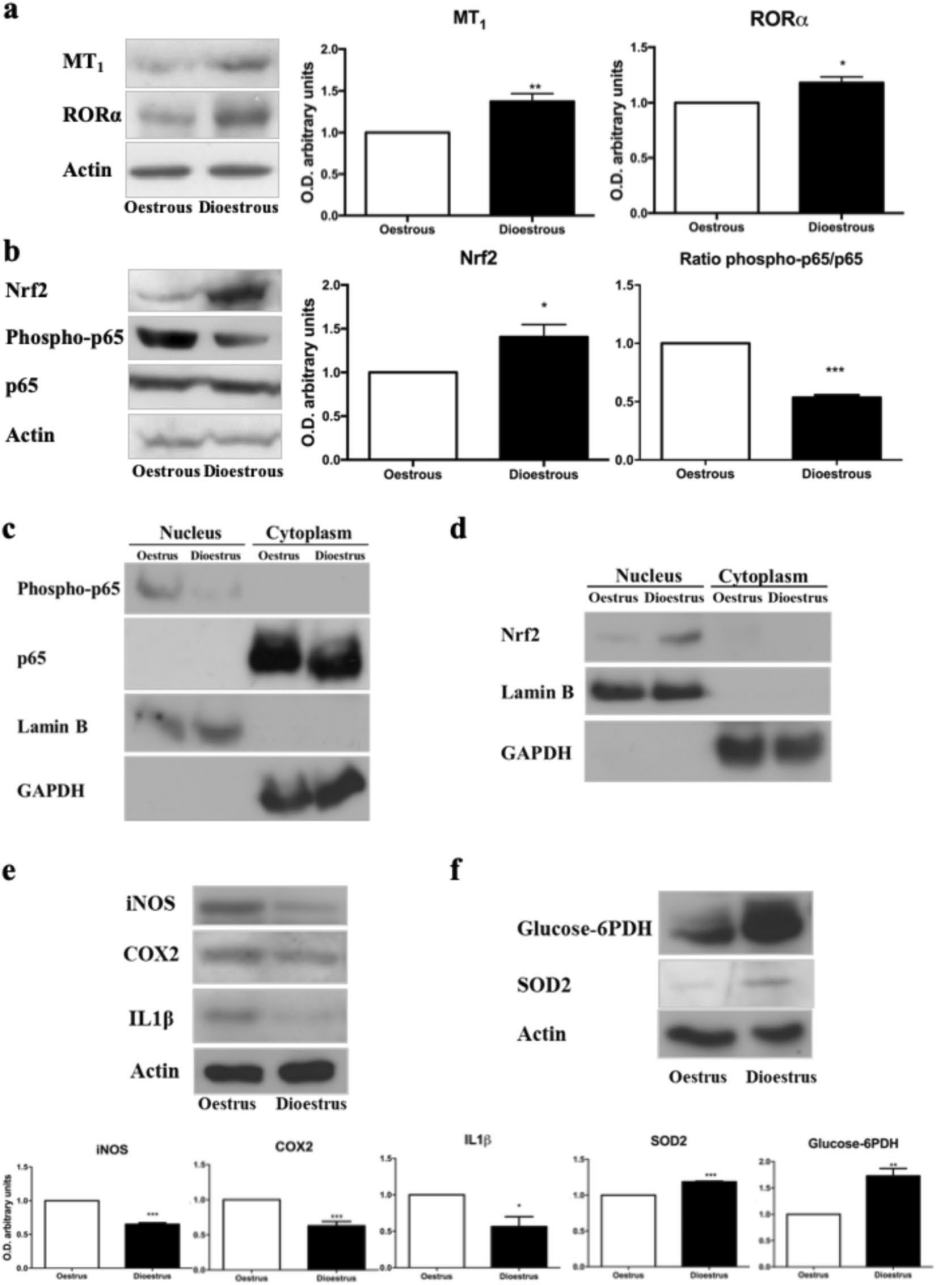
Melatonin role in the cellular response to oxidative stress. (**a**) MT-1, RORα and (**b**) Nrf2, phospo-p65 and p65 protein band intensities in the different fractions of the Harderian glands from the oestrus and dioestrus phases. The bar graphs of MT-1 and RORα (**a**) and Nrf2 (b) quantifies the optical densities of the western blot bands normalized to ß-actin and the phospho-p65/p65 ratio (**b**) as NF-κB activation marker. (**c**) Phopho-p65, p65 and (**d**) Nrf2, Lamin B and GAPDH protein band in nuclear and cytosolic fractions from the oestrus and dioestrus phases. (**e**) iNOS, COX2, IL1β and (**f**) Glucose-6PDH and SOD2 protein band intensities in the different fractions of the Harderian glands from the oestrus and dioestrus phases. The bar graphs of iNOS, COX2, IL1β (**e**) and Glucose-6PDH and SOD2 (**f**) quantifies the optical densities of the western blot bands normalized to ß-actin. Data are expressed as the means ± SEM and are calculated from at least three separate experiments. *(p < 0.05), **(p < 0.01), ***(p < 0.001).

The role of melatonin in oestrous cycle physiology can be assessed by the expression of melatonin receptors, which have been described in HG^26^. MT_1_ is a G protein-coupled receptor at the plasma membrane, and it is activated by the pineal-synthesized melatonin^26^. RORα is a nuclear melatonin receptor that plays an important role in cell protection against oxidative stress^27^.Western-blot analyses for MT_1_ and RORα proteins showed higher expression levels of both in dioestrus (Fig. 1a, p < 0.05).

Nrf2 is an essential transcription factor in the cellular response to oxidative stress^28^. Under basal conditions, nuclear levels of Nrf2 remain relatively low because it is sequestered in the cytosol^19,28^. High oxidative stress levels activate the canonical Nrf2 pathway^29^. Our study reveals a strong Nrf2 activation in the dioestrus phase, compared to the one in oestrus phase. Nrf2 showed higher expression both in total homogenates (Fig. 1b, p < 0.05) and in nuclear fractions (Fig. 1d). To further analyse Nfr2 activation, Glucose-6Phosphate Dehydrogenase (Glucose-6PDH) and SOD2 levels were tested. Both proteins also showed higher expression in dioestrus (Fig. 1f, p < 0.01).

Nuclear factor-kappa B (NF-κB) is a transcription factor that also plays a key role in cell responses to oxidative stress^30^. We examined its activation state using immunoblot analysis of key proteins in the NF-κB pathway: p65 and phospho-p65 (Ser536). Activation occurs via phosphorylation of p65 at Ser536, and only the phosphorylated protein has nuclear localization and transcriptional activity^4^. Total amount of p65 did not show significant differences between phases of the oestrous cycle of HG (Fig. 1b). However, phosphorylated p65 expression was significantly lower in dioestrus than oestrus phase (Fig. 1b). Thus, to characterize the NF-κB activation, we evaluated the ratio of phospho-p65 respect to total p65 protein and confirmed a lower level of phospho-p65/p65 in dioestrus compared to oestrus (Fig. 1b, p < 0.001). Oestrus and dioestrus nuclear fractions were evaluated to test phopho-p65 translocation to the nucleus, lower expression of phopho-p65 was found in dioestrus (Fig. 1c). Then, downstream proteins were evaluated: iNOS, COX2 and IL1β, being last 2 proteins also pro inflammatory enzymes. All of them showed lower expression in the dioestrus phase (Fig. 1e, p < 0.05). Our data suggest a lower activation level of the NF-κB pathway in the dioestrus phase.

In order to further understand the impact of NF-κB on autophagy, p50^KO^ (NF-κB1^KO^) and wild type (WT) mouse embryonic fibroblasts (MEFs) were used and p62 expression was examined. Mutant cells showed a reduction of p62 expression (Fig. S1). Furthermore, proteins degraded by selective autophagy: SDHB or complex II for mitophagy and Plin2 and Plin3 for lipophagy, were actively degraded in WT cells while accumulated in p50^KO^ ones. These results would indicate that deficient NF-κB signalling have a negative impact on autophagy.

### Functional mitochondrial status

**Figure 2:**
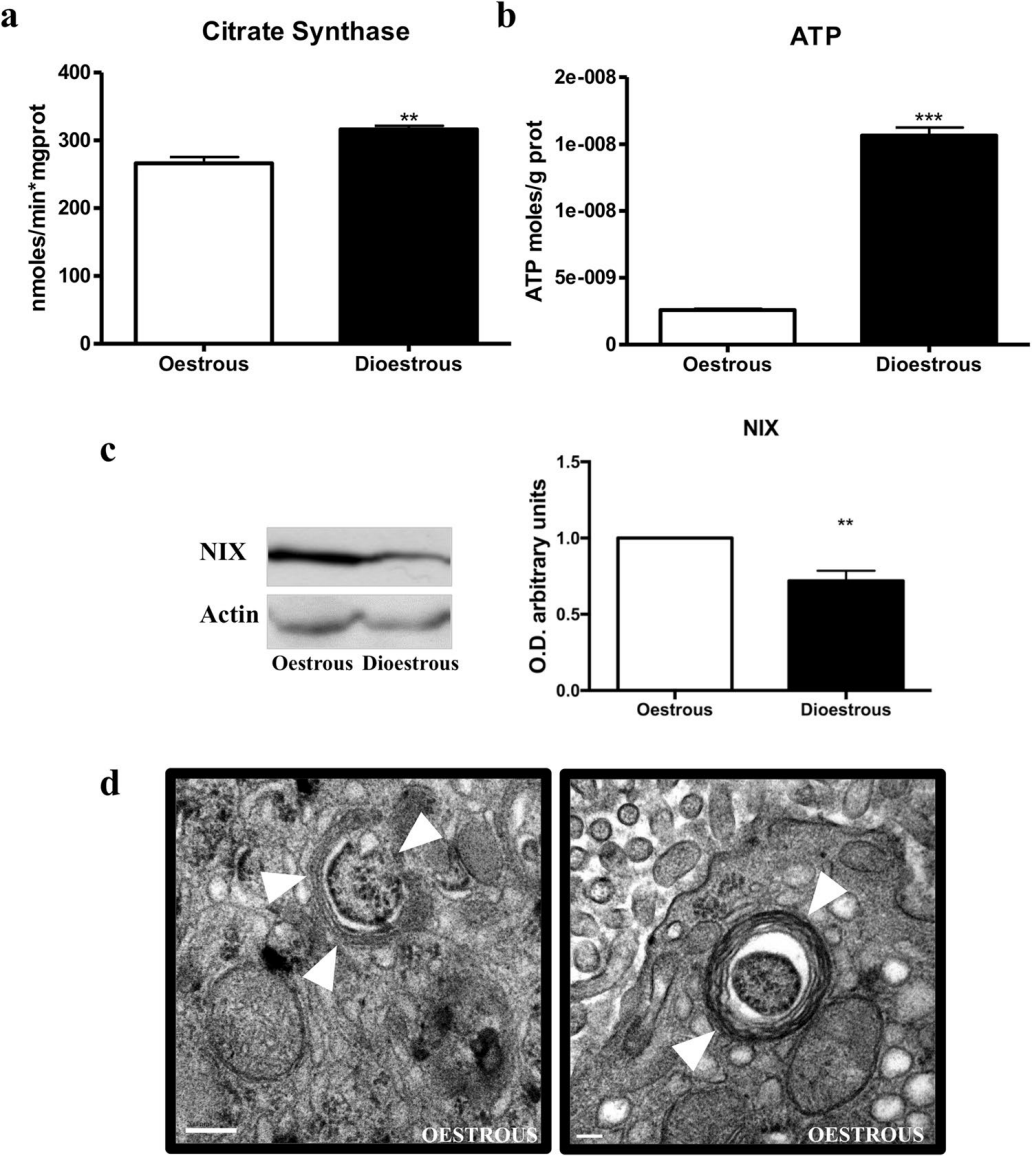
Mitochondrial status and clearance. Mitocondrial (**a**) Comparison of Citrate Synthase (CS) activity, which results are expressed in nmoles/min*mg protein. (**b**) ATP intracellular content, which results are expressed as ATP moles/g protein. (**c**) NIX protein band intensity and NIX bar graph that quantifies the optical densities of the western blot bands normalized to ß-actin. Data are expressed as the means ± SEM and are calculated from at least three separate experiments. *(p < 0.05), **(p < 0.01), ***(p < 0.001). (**d**) Electron micrographs of cells in oestrus phase showing mitochondria within an autophagosome (arrowhead) as representative images. Similar results were obtained from three separate experiments. Scale bar, 0.2 µm.

Mitochondria play an essential role in cell survival^31^, but their activity generates free radicals that alter the oxidative balance^32^. Mitochondria also play a key role in lipid metabolism^33^. To characterize mitochondrial functional status, we studied citrate synthase activity and ATP generation. Citrate synthase is a mitochondrial matrix enzyme, essential in Krebs cycle that is an indicator of healthy mitochondrial population^34^. Based on this mitochondrial marker, the number of mitochondria is higher in dioestrus than in oestrus (Fig. 2a, p < 0.05). Accordingly, ATP levels were also higher in dioestrus, indicating better mitochondrial efficiency (Fig. 2b, p < 0.001).

Mitophagy, the selective autophagy pathway to degrade defective mitochondria, was also assessed. The mitochondrial protein NIX has been described as an autophagy receptor, mediating the clearance of damaged mitochondria^35^. NIX expression was lower in dioestrus than oestrus (Fig. 2c, p < 0.01). Using electron microscopy (EM) we corroborated the elevated presence of mitophagosomes during oestrus (Fig. 2d). Mitochondria are healthier in dioestrus, hence, their selective degradation through autophagy is less prominent during this phase.

### Lipophagy

**Figure 3:**
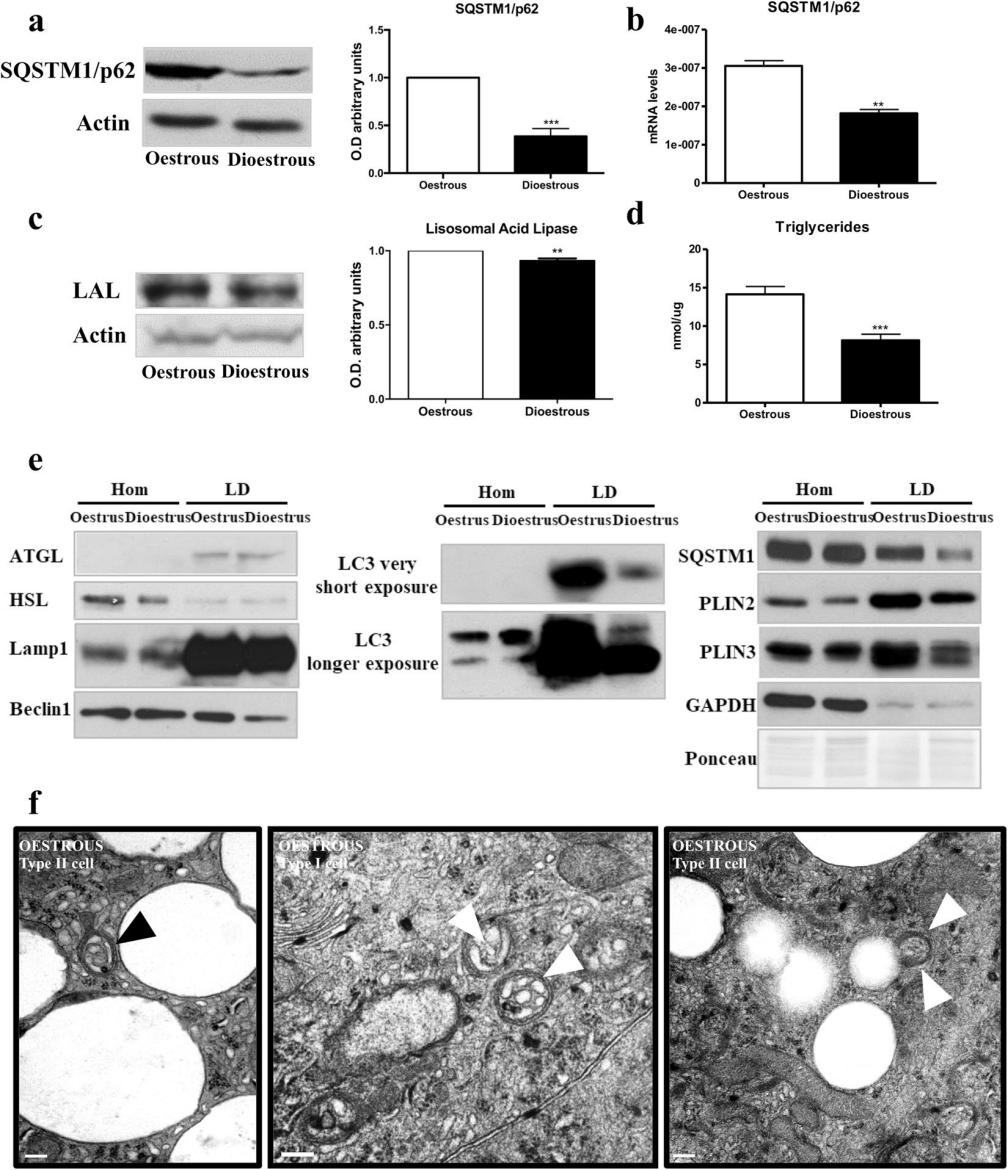
Lipolisis-related autophagy activity (**a**) SQSTM1/p62 and (**c**) LAL protein band intensities and its respective bar graphs that quantifies the optical densities of the western blot (WB) bands normalized to ß-actin. (**b**) SQSTM1/p62 mRNA expression quantification, normalized to 18s. (**d**) Triglyceride levels in oestrus and dioestrus, results are expressed in nmoles/μg protein. (**e**) WB for indicated proteins in total homogenates (Hom) and lipid droplets (LD). Data are expressed as the means ± SEM and are calculated from at least three separate experiments, with each experiment performed in triplicate. *(p < 0.05) **(p < 0.01) (**f**) Electron micrographs of cells in oestrus phase showing lipid droplets within autophagosomes (arrowheads). Scale bar, 200 nm.

The LC3-interacting protein SQSTM1/p62 is a key autophagic protein for cell homeostasis mediating the selective specific degradation of protein aggregates and cytoplasmic bodies^36^. Recent studies have described SQSTM1/p62 as a key mediator in lipolysis^37^ and also, in lipophagy^38,39^. SQSTM1/p62 protein (Fig. 3a, p < 0.01) and RNA (Fig. 3b, p < 0.01) levels were significantly lower during the dioestrus.

Lipolytic processes were studied by assaying the expression of lysosomal acid lipase (LAL) and the triglyceride (TG) levels. LAL is involved in the degradation of cholesteryl esters and triglycerides^40^. Our data showed LAL lower expression in the dioestrus phase (Fig. 3c, p < 0.05). Concomitantly, TGs levels were also lower in the dioestrus phase (Fig. 3d, p < 0.001). LAL degrades the lipids provided by autophagy machinery (lipophagy) in the lysosome^41^. To characterize the level of lipophagy, we quantified the presence of the autophagy machinery components in the LD in both phases. First, we isolated LDs from HGs at oestrus and dioestrus, the expression of cytosolic lipases (ATGL and HSL) was assessed to evaluate classical lipolysis, no changes were found in the LD isolations. Then, we determined the presence and expression levels of autophagic proteins as Lamp1, Beclin, LC3-II, SQSTM1/p62, Plin2, Plin3 and, also, GAPDH to exclude cytosolic contamination^42^, by western blot. The expression of all the autophagic proteins was lower in LDs from the dioestrus phase while the total homogenate (Hom) was unchanged (Fig. 3e). This result indicates lower LD degradation in dioestrus than oestrus phase. Furthermore, our results from EM analysis of HGs during the oestrus cycle revealed the presence of lipid droplets inside the autophagosomes showing the high levels of lipid degradation (Fig. 3f). In order to further quantify the abundance of LDs we analyse the presence of the perilipin (Plin) family proteins that coats LDs^43^. As expected, we observed lower expression of Plin2 and Plin3 in dioestrus, indicating fewer LDs in this phase (Fig. 3e).

**Figure 4:**
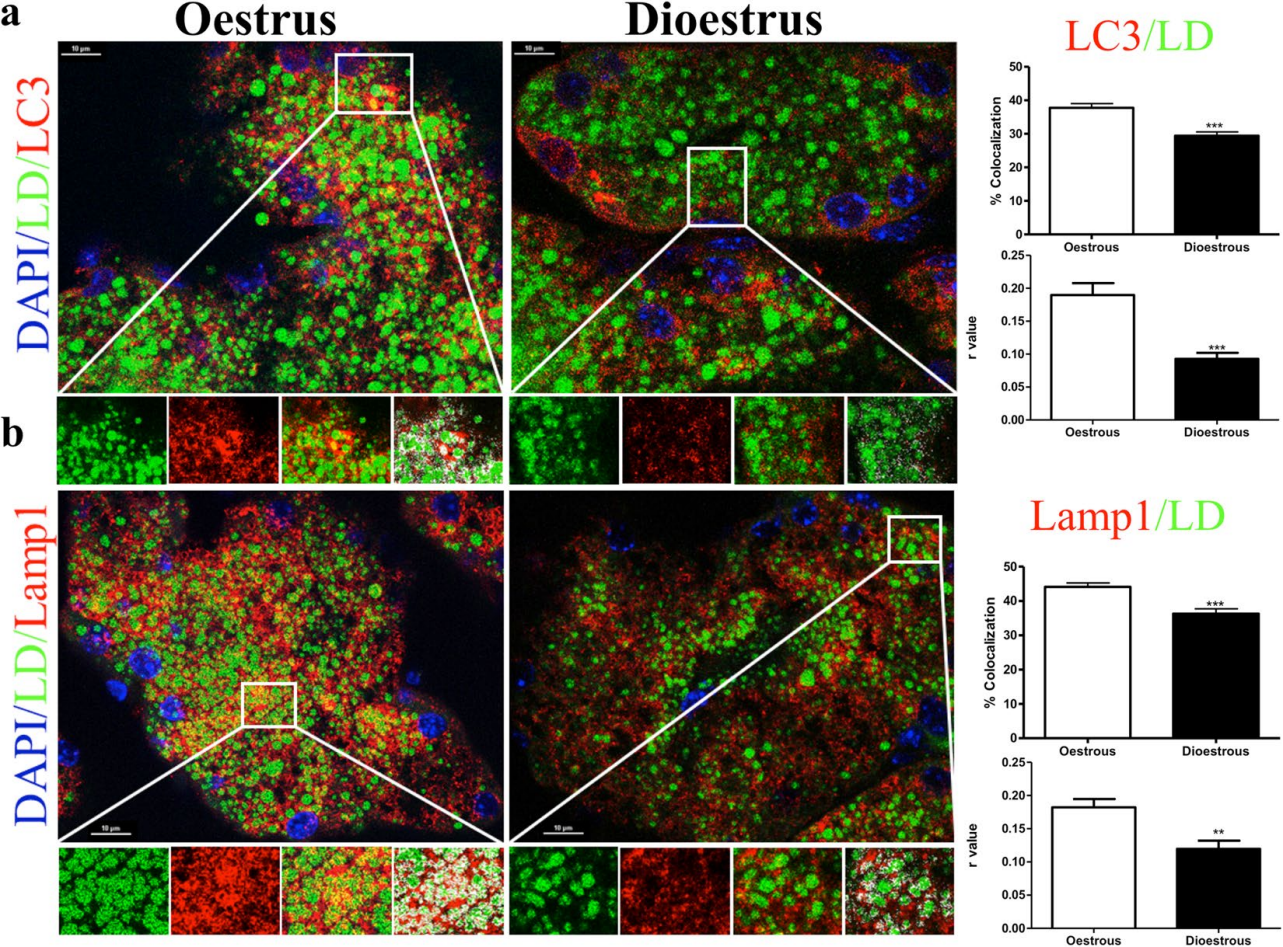
Differential lipophagy activation during the estrous cycle. Immunofluorescence for LC3 and BODIPY 493/503 (**a**) and Lamp1 and BODIPY 493/503. Colocalization determined by the “colocalization finder” plugin in ImageJ is shown in white in the fourth enlarged panel. Enlarged image scale bar: 10 μm. (**b**) and its respective bar graphs that quantifies the percentage of colocalization.

We then use immunohistochemistry to confirm the previous results and determine whether LC3 (Fig. 4a) and Lamp1 (Fig. 4b), autophagosome and lysosomal markers respectively, co-localize with BODIPY, a dye that stains neutral lipids in the LD. We found less colocalization (through both Manders and Pearson tests) of both markers with the LDs in dioestrus phase (Fig. 4a,b, p < 0.001), supporting the idea of higher lipophagy levels during oestrus.

## Discusion

How sexual hormones modulate selective autophagy is poorly understood, this lack in knowledge aggravates the sexual bias we could easily find in biological research. Harderian gland (HG) has an intrinsic plasticity to quickly respond to several internal and external stimuli via both morphological and biochemical changes^44^. We have previously shown that female HG variations are dependent on the fluctuations in sex hormones during the oestrous cycle, which makes HG the perfect model to understand how these hormonal changes modulate autophagy. These autophagic changes during oestrous cycle are conserved among rodents^45^.

Melatonin is a hormone secreted by the pineal gland, usually related to protection against oxidative stress due to its antioxidant properties^23^. The rate of melatonin secretion varies synchronously with the vaginal cycle, being higher when the oestrogen levels are lower at dioestrus^24^. It has been described that melatonin displays an anti-estrogenic effect^25^ mediated by the membrane-bound receptor MT_1_^46^, although the contribution of nuclear receptors is still unclear^46^. Accordingly, melatonin receptors, both MT_1_ (plasma membrane) and RORα (nucleus), are more abundant in dioestrus. Thus, the increased melatonin signalling, via its receptors, may be involved in reduction of pro-inflammatory mediators and alleviating the higher oxidative stress levels that exist during the oestrus phase^12^. In its role as antioxidant, melatonin has been described as inductor of Nrf2 activation, enhancing its nuclear translocation and subsequent antioxidant response (ARE) binding^19,47^. This translocation process has been observed in our study under dioestrus phase and an increase in antioxidant protein expression is also seen in dioestrus phase, what leads us to assume that melatonin antioxidant effect in the HG is mediated by its receptors and potentiated by Nrf2 activation in this phase along with minimal oestrogen levels.

The transcription factor NF-κB is a pro-inflammatory factor activated by oxidative stress and inhibited by melatonin^19^. Here we have shown that NF-κB is activated during oestrus phase along with high expression of pro inflammatory factors (COX2 and IL1β) and elevated levels of oxidative stress. Thus, activation of NF-κB favour the pro-inflammatory profile in HG at the oestrus phase. It has been also shown that NF-κB promotes mitophagy by SQSTM1/p62 induction^48^ and our analysis corroborates this role (Fig. S1). Accordingly, in our results female HG showed activation of SQSTM1/p62 in oestrus phase. Moreover, regarding mitochondrial clearance, the NIX protein, which is localized in the mitochondrial outer membrane, has been defined as a mitochondrial receptor for mitophagy^35^. Our present results showed a higher NIX expression in the female HG at the oestrus phase than in the dioestrus one. Additionally, we have observed the presence of mitophagosomes by EM (Fig. 2d). All together these data show the activation of mitophagy in the female’s HG during oestrus mediated by NF-κB activation. Moreover, mitochondrial depolarization, tested with Citrate synthase activity, and the subsequent mitochondrial ROS generation have been previously linked to NIX ability to directly activate the autophagy machinery via mTOR inhibition^49^. Consistent with these markers and data, mitochondrial energetic functions, assayed by ATP concentration, were significantly lower in the HGs during the oestrus phase. Therefore, increased levels of the mitophagy marker NIX, formation of mitophagosomes, and impaired mitochondrial functionality suggest an intense mitochondrial removal by selective autophagy in the female HG at the estrus phase promoted by NF-κB activation.

The role of autophagy in lipid metabolism has been recently discovered and lipophagy has been shown as the main mechanism to mobilize lipid stores^15^. Lipophagy was first discovered in liver and, currently, is described in many other tissues^14^. SQSTM1/p62 participates in the selective removal of organelles, as mitochondria or LDs through autophagy^38,39^. SQSTM1/p62 and other autophagic machinery directly contributes to the mobilization of lipids from LDs to lysosomes, where they get degraded by lipases^14^. Our data showed higher lipolytic activity in oestrus phase due to the higher expression of lysosomal acid lipase (LAL). Accordingly, autophagy and lysosomal proteins, but not cytosolic lipases, were accumulated in the LDs in this phase, showing higher lipophagy activity. Furthermore, our EM studies revealed the presence of LDs within the autophagosomes. Perilipins showed less expression in dioestrus, indicating lower number of LDs. Thus, our data relate lipolytic and lipophagy activities with the cellular changes usually observed in the HG during the oestrous cycle and, until now, not well understood. The higher lipophagy activity in oestrus could be the process behind the Type II cells disappearance.

**Figure 5:**
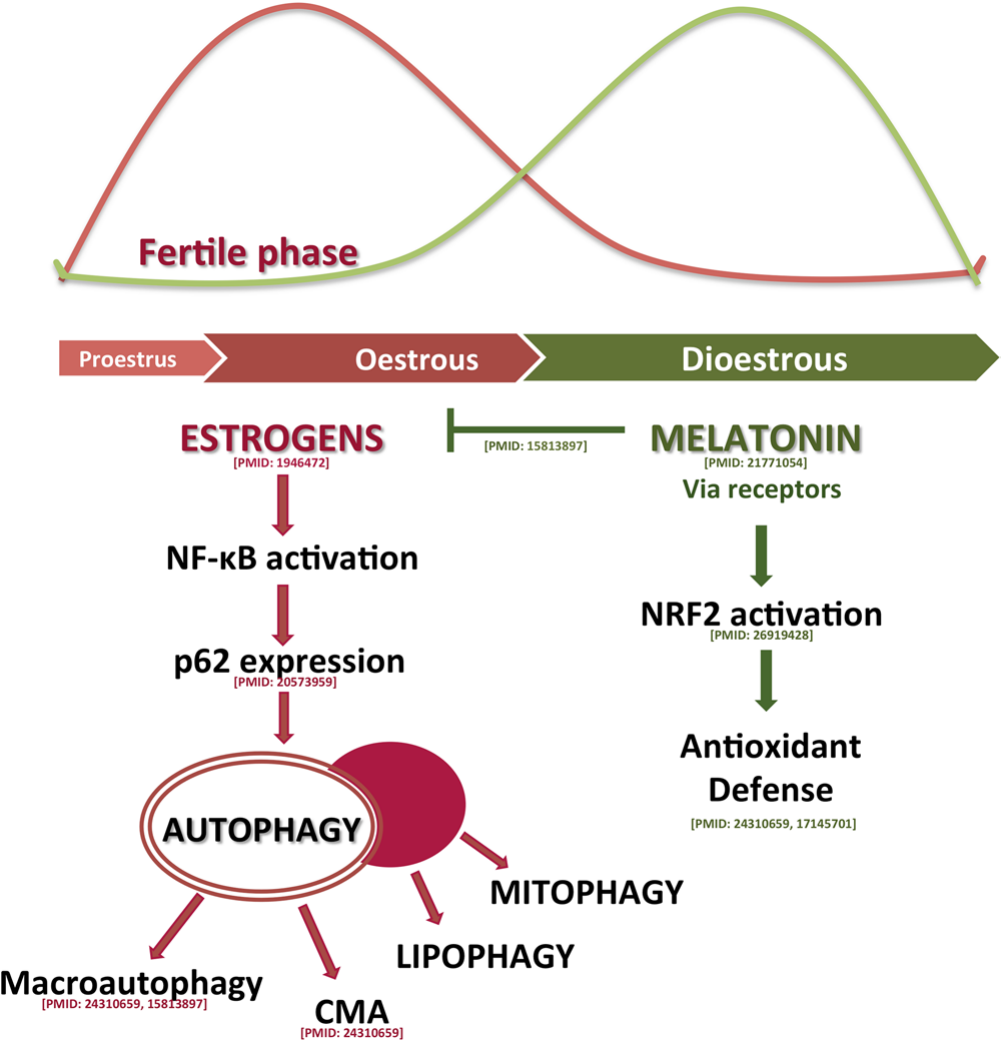
Modulation by melatonin of the estrous cycle mechanisms. The scheme proposes that oxidative stress is one of the key processes involved in the estrous cycle with melatonin as modulator of it.

We have shown that the HG in the oestrous cycle is characterized by important variations in oestrogen levels and oxidative stress (Fig. 5), where melatonin may be its primary moderator, in basis of its clear relationship with both factors: it is a well-known antioxidant and has important antiestrogenic effects. Melatonin interacts with 2 of the oestrogen-signalling pathways and it decreases circulating levels of oestradiol^50,51^. Melatonin levels are reduced in the oestrus phase and entail the activation of NF-κB and the elevated oxidative stress, which induces not only macroautophagy and CMA^12,52,53^ but also increases other selective autophagic processes. All associated to a decrease in mitochondrial activity, leading to degradation via mitophagy. Likewise, activation of NF-κB may also lead to lipolytic processes, as suggested by the increase of the autophagic and lysosomal proteins in the LDs and the degradation of large lipid droplets in Type II cells. These degradation processes require a high lysosomal effort, as shown by the increases in LAL expression. Melatonin, via both membrane and nuclear receptors, reduces pro-inflammatory mediators and enhances the expression of Nrf-2 in dioestrus phase. Consequently, autophagy is blocked, and porphyrin release is reduced, and the gland returns to a rest period.

## Materials and Methods

### Animals

Eight-week-old female Syrian hamsters (*Mesocricetus auratus*) (Harlan Interfauna Ibérica, Barcelona, Spain) were housed 2 per cage under long days with a 14:10 light:dark cycle (lights on daily from 07:00 to 21:00) at 22 ± 2 °C (n = 8 per experimental condition). Hamsters received water and a standard pellet diet *ad libitum*. The Oviedo University Local Animal Care and Use Committee approved the experimental protocols (reference 33443591). All experiments were carried out according to the Spanish Government Guide and the European Community Guide for Animal Care (Council Directive 86/609/EEC).

All animals were monitored daily by vaginal smears to determine their reproductive phase (proestrus, oestrus, metoestrus, dioestrus) according to the method of Orsini^54^, during 3 consecutive cycles. For studying opposite phases, we collected females during the oestrus and dioestrus phases because these phases present the highest plasmatic differences in oestrogen concentration during the oestrous cycle^55^. After determination of the specific oestrous phase, animals were sacrificed, and the hamster Harderian glands (HGs) were immediately removed, frozen in liquid nitrogen, and stored at −80 °C until performing of the experiments, HGs were used directly for lipid droplet isolation.

### Isolation of proteins

HGs (0.1 g) were homogenized using a Polytron homogenizer at 4 °C in 1 ml of lysis buffer (50 mM Tris/HCl, 150 mM NaCl at pH 7.4 and protease and phosphatase inhibitors). The tissue homogenates were then centrifuged for 6 min at 3000 rpm at 4 °C. The supernatants were collected and centrifuged again under the same conditions. Nuclear and cytoplasmic fractions were prepared using the sucrose gradient method^4^. The protein concentration of the supernatants was measured by the method of Bradford^56^.

### Isolation of lipid droplet (LD) fractions

LD fraction from Harderian gland was isolated following the protocol for brown adipose tissue with few alterations^42^. Tissues were homogenized in 0.25 M sucrose and centrifuged at 6,800 g for 5 min/4 °C. Supernatants including the fatty layer were centrifuged at 17,000 g/10 min/4 °C to eliminate unwanted cellular fractions. Supernatant from the 17,000 g spin was adjusted to 20% sucrose and centrifuged in a discontinuous sucrose density gradient at 27,000 g for 30 min at 4 °C. LD fractions were delipidated using successive washes in acetone and ether and solubilized in 2% SDS for immunoblotting.

### Immunoblotting

Protein samples were prepared in western-blotting sample buffer (65.8 mM Tris-HCl, pH 6.8, 2.1% SDS, 26.3% (w/v) glycerol, 0.01% Bromophenol Blue). SDS-polyacrylamide gels were run and analysed as previously described^23,34^. Primary antibodies applied were as follows: nuclear factor erythroid 2-related factor 2 (Nrf2), superoxide dismutase 2 (SOD2), retinoid-related orphan receptor alpha (RORα) and melatonin receptor 1A (MT1) from Santa Cruz Biotechnology (Santa Cruz, CA, USA), adipose triglyceride lipase (ATGL), beclin 1, cyclooxygenase-2 (COX2), interleukin 1β (IL1β), hormone-sensitive lipase (HSL), lamin B, microtubule-associated protein 1A/1B-light chain 3 (LC3), nuclear factor kappa-B1 p50 subunit (NF-κB1 p50), nuclear factor kappa-B p65 subunit (NF-κB p65), nuclear factor kappa-B p65 subunit phosphorylated at Serine 536 (Phospho-NF-κB p65 [Ser536]) and sequestosome-1 (SQSTM1/p62) from Cell Signaling Technology (Boston, MA, USA), glyceraldehyde 3-phosphate dehydrogenase (GAPDH) and lysosomal acid lipase (LAL), inducible nitrate synthase (iNOS) and succinate dehydrogenase B (SDHB) antibody from Abcam (Cambridge, UK), lysosomal-associated membrane protein 1 (Lamp1) from Developmental Studies Hybridoma Bank (Iowa City, Iowa, USA); Bcl2/Adenovirus E1B 19 kDa and protein-interacting protein 3-like (BNIP3L/NIX) and glucose 6-phosphate dehydrogenase (Glucose-6PDH) from Sigma-Aldrich (St. Louis, MO, USA), perilipin (PLIN) 2 (Progen Biotechnik, Heidelberg, DE) and PLIN3 (ProSci Inc, Poway, CA, US). Primary antibodies were mostly diluted 1:1000 in blocking buffer, except for Phospho-NF-κB p65 (Ser536), which was diluted 1:500, and Nrf2, which was diluted 1:250. Goat β-actin antibody (Santa Cruz Biotechnology, Inc.) diluted at 1:1000 was always assayed as a loading reference. All the primary antibodies have been previously validated^4,12,23,57^. After washing in TBS-T (20 mM Tris-HCl, 150 mM NaCl, pH 7.4 and 0.05% Tween-20), the membranes were then incubated with the corresponding horseradish peroxidase-conjugated secondary antibody: anti-rabbit for most primaries, but anti-mouse for β-actin, or anti-rat for Lamp1 or anti-guinea pig for the PLIN2 (Santa Cruz Biotechnology, Inc.) diluted 1:2500. Binding of antibodies to their antigens was detected using the Western Blotting Luminol Reagent (sc-2048; Santa Cruz Biotechnology, Inc.) according to the manufacturer’s protocol. Negative controls were performed with either no primary or no secondary antibodies. No bands were detected in any case.

The results were calculated from at least three separate experiments for each antibody and were normalized to actin. Band intensity was quantified using the Quantity One 1D analysis software v. 5.5.1. (Bio-Rad Laboratories Inc., Hercules, CA, USA). Then dioestrus values were normalized to oestrus values in order to make comparisons.

All the original blots are shown in the Supplementary Fig. 2.

### Fluorescence microscopy

HGs were fixed overnight in 4% paraformaldehyde (PFA) solution at 4 °C. After three washes in PBS, HGs were cryoprotected first overnight at 4 °C in 15% sucrose and then 4 hours at room temperature in 30% sucrose. HGs were oriented and embedded in OCT and then frozen in liquid nitrogen. 10 μm sections were obtained in a cryostat. Sections were rinse in PBS and then blocked with 3% horse serum, 1% BSA in 1x PBS containing 0.4% triton X-100 for 1 hour at room temperature, then they were incubated with primary were incubated with primary antibodies Lamp1 (Developmental Studies Hybridoma Bank, Iowa City, Iowa, USA) and LC3 (Cell Signalling Technology, Boston, MA, USA) and secondary antibodies anti-rat and anti-rabbit, respectively (Alexa Fluor 647 conjugated, Invitrogen (Carlsbad, Ca, USA)). For lipid droplet (LD) detection, sections were incubated with BODIPY 493/503 for 20 min at RT (Molecular Probes Inc., Eugene, OR, USA). Mounting medium contained DAPI (4′,6-diamidino-2-phenylindole) to visualize the nucleus (Molecular Probes Inc., Eugene, OR, USA). Negative controls were performed with either no primary or no secondary antibodies. No staining was detected in any case. Images were acquired on a Nikon A1R confocal inverted (Nikon Instruments INC., NY, USA) using X100 objective/1.4 numerical aperture. Images were acquired at similar exposure times in the same imaging session. Image slices of 0.2 µm thickness were acquired and deconvolved using the Huygens (Huygens Essential, Hilversum, The Netherlands) acquisition/analysis software. Quantification was performed in deconvolved images after appropriate thresholding using the ImageJ software (NIH)^58^ in a minimum of 30 acini from at least 3 experiments. Cellular fluorescence intensity was expressed as mean integrated density as a function of individual cell size. Percentage colocalization was calculated using the JACoP plugin in single Z-stack sections of deconvolved images (Manders and Pearson test). Colocalization is shown in native images and/or as white pixels using the “colocalization finder” plugin in ImageJ, using same threshold for all the images^58^.

### Reverse transcription (RT)

Total RNA was extracted using the Tripure™ Isolation Reagent (Roche Applied Science, Mannheim, Germany), according to the manufacturer’s instructions. The yield of total RNA was determined by measuring the absorbance (260/280 nm) using a NanoDropND-1000 spectrophotometer (Nano-Drop Technologies, USA). RT was completed with the high-capacity cDNA Reverse Transcription Kit (Applied Biosystems, Foster City, CA, USA), following manufacturer instructions. Reactions were performed for 10 min at 25 °C, 2 h at 37 °C and terminated by heating for 5 sec at 85 °C. The reaction mixture was maintained at −20 °C until further use.

### Quantitative real-time PCR

Quantitative real-time PCR of the different mRNAs was performed in triplicate using gene-specific primers and SYBR® Green. Oligonucleotide primers were designed using Primer Express 2.0 software (Applied Biosystems, Foster City, CA, USA). The primer sequences and corresponding GenBank accession numbers are given in Table 1. As an internal control for normalization, PCR reactions were performed concurrently with the amplification of a reference gene, 18 S ribosomal RNA (rRNA) that proved to be stable in all the conditions studied.

**Table 1:** Real time PCR primers the GenBank accession numbers.

Target gene	Forward primer (5′-3′)	Reverse primer (5′-3′)	Genbank
18S	GATTAAGTGCCCTTTGTA	GATCCGAGGGCCTCACTAAAC	v01270
p62 (Sequestosome 1)	CCATGGGTTTCTCGGATGAA	ATCCAGTATTCAAAGCACCCTCC	NM_175843.3

Real time-PCR was performed on an Step-one plus (Applied Biosystems, Foster City, CA, USA) real-time thermal cycler using the SYBR® Green PCR Master Mix kit (Applied Biosystems, Foster City, CA, USA) with the following thermal cycler settings: one cycle of 10 min at 95 °C, 40 cycles of 15 s at 95 °C and 1 min at 60 °C. Cycle thresholds for both genes were selected immediately above the baseline and within the linear range on log scaling. Each reaction (20 μl) consisted of a 2 μl cDNA aliquot, 300 nM of each primer and 10 μl of SYBR® Green PCR Master Mix containing AmpliTaq gold DNA polymerase. Increases in the amount of SYBR® Green reporter dye fluorescence during the amplification process were analysed with Sequence Detector software (SDS version 1.6 Applied Biosystems, Foster City, CA, USA). Relative change in expression of the target genes was determined by the following equation:

$${\rm{Fold}}\,{\rm{change}}=2-\Delta \mathrm{Ct},\,\Delta \mathrm{Ct}=({\rm{Ct}}\,{\rm{target}}-{\rm{Ct}}\,18{\rm{S}}\,{\rm{ribosomal}}\,{\rm{RNA}}\,({\rm{rRNA}})).$$

The Ct value is the cycle number at which the fluorescence signal crosses the designated threshold^59^.

### Morphological studies

For ultrastructural studies, HGs were treated as previously described^4^. HGs were lightly fixed by immersion in a solution containing 1.5% glutaraldehyde and 2.5% paraformaldehyde in 0.1 M phosphate buffer (pH 7.4). Fixation was continued overnight at 4 °C using fresh fixing solutions. Tissues were then postfixed in 1% Osmium (OsO4) for 2 hours. After dehydration in a graded acetone series, the tissue fragments were embedded in the epoxy resin TAAB 812, and 1 μm semithin sections were stained with toluidine blue. Ultrathin sections were collected on copper grids, stained with uranyl acetate-lead citrate, and examined using a Zeiss EM-109 transmission electron microscope (Zeiss, Oberkochen Germany) operating at 80 kV.

### Mitochondrial functionality

As an indicator of the mitochondrial population, citrate synthase (CS) activity was determined spectrophotometrically at 412 nm and 30 °C, as previously described^34^.

The intracellular ATP content in the HGs was measured with an Adenosine-5′-triphosphate bioluminescent assay kit (FL-AA, Sigma Aldrich Inc.). Bioluminescent luciferase-luciferin reactions provide the basis of simple, rapid, and highly sensitive assays for ATP^60^. Samples (100 μL) of tissue homogenates diluted 1:100 were mixed with 100 μL of ATP assay mix dilution buffer FL-AAB (pH 7.8). Light production was then immediately measured by luminescence using a Luminometer Turner Designs TD-20/20 (Turner BioSystems Inc., Sunnyvale, CA, USA)^61^.

### Triglyceride levels

The triglyceride (TG) levels were determined using a triglyceride quantification kit following the manufacturer’s indications (Abcam, Cambridge, UK). In this assay, triglycerides were converted to free fatty acids and glycerol. The glycerol was oxidized and combined with a probe to generate the luminometric signal. HG gland samples were lysed using NP-40 solution, then incubated with the lipase for 20 minutes. A final incubation of 1 hour with the Triglyceride reaction to ensure the oxidation of the glycerol and the colorimetric reaction. Measurement was performed at OD 570 nm in a Varioskan Flash (Thermo Fisher, Waltham, MA, USA).

## Supplementary information

Supplementary information

## Data Availability

The authors declare that the data supporting the findings of this study are available within the paper.
