# Supplementary material for: Selective autophagy, lipophagy and mitophagy, in the Harderian gland along the oestrous cycle: a potential retrieval effect of melatonin
Source: Sci Rep. 2019 Dec 9;9:18597. doi: 10.1038/s41598-019-54743-5 (PMC6901547; doi:10.1038/s41598-019-54743-5)
Supplement: Supplementary file 1 — Supplementary information [file 41598_2019_54743_MOESM1_ESM.pdf]

SI: Selective autophagy, lipophagy and mitophagy, in the Harderian gland along the oestrous cycle: a potential retrieval effect of melatonin.

Marina García-Macia[1#*], Adrián Santos-Ledo[2*], Beatriz Caballero[3], Adrián Rubio-González[3], Beatriz de Luxán-Delgado[3], Yaiza Potes[3], Susana Rodríguez-González[3], José Antonio Boga[4], Ana Coto-Montes[3#]

SUPPLEMENTARY MATERIALS AND METHODS

In vitro experiments

Passage 10 wild type (WT) and p50^{KO} mouse embryonic fibroblasts (MEFs) were grown in high glucose (4.5 g/l), glutamine supplemented DMEM (Invitrogen, Carlsbad, CA, USA) with 10% fetal bovine serum (FBS) (Invitrogen) and antibiotics. Oleic acid (250mM) was used for inducing lipid droplet formation, mimicking the oestrus phase. Then cells were transferred to serum-free DMEM to induce lipophagy. Flux assays were used to quantify autophagy activity. Accumulation of autophagy substrates, p62, SDHB PLIN2 and PLIN3, in the presence of inhibitors of lysosomal proteolysis, ammonium chloride (20 mM) and leupeptin (100 μM), reflects autophagy activity. Briefly, cells were cultured in the presence or absence of lysosomal inhibitors for 2 h following which, cells were collected and lysed and subjected to immunoblotting for p62, SDHB PLIN2 and PLIN3.

FIGURE LEGENDS

Supplementary Figure 1. The lack of p50 (NFκB1) disturbs autophagy in vitro. Indicated protein band intensities in the different fractions of the Harderian glands from the oestrus and dioestrus phases. The bar graphs quantify the optical densities of the western blot bands normalized to ß-actin.

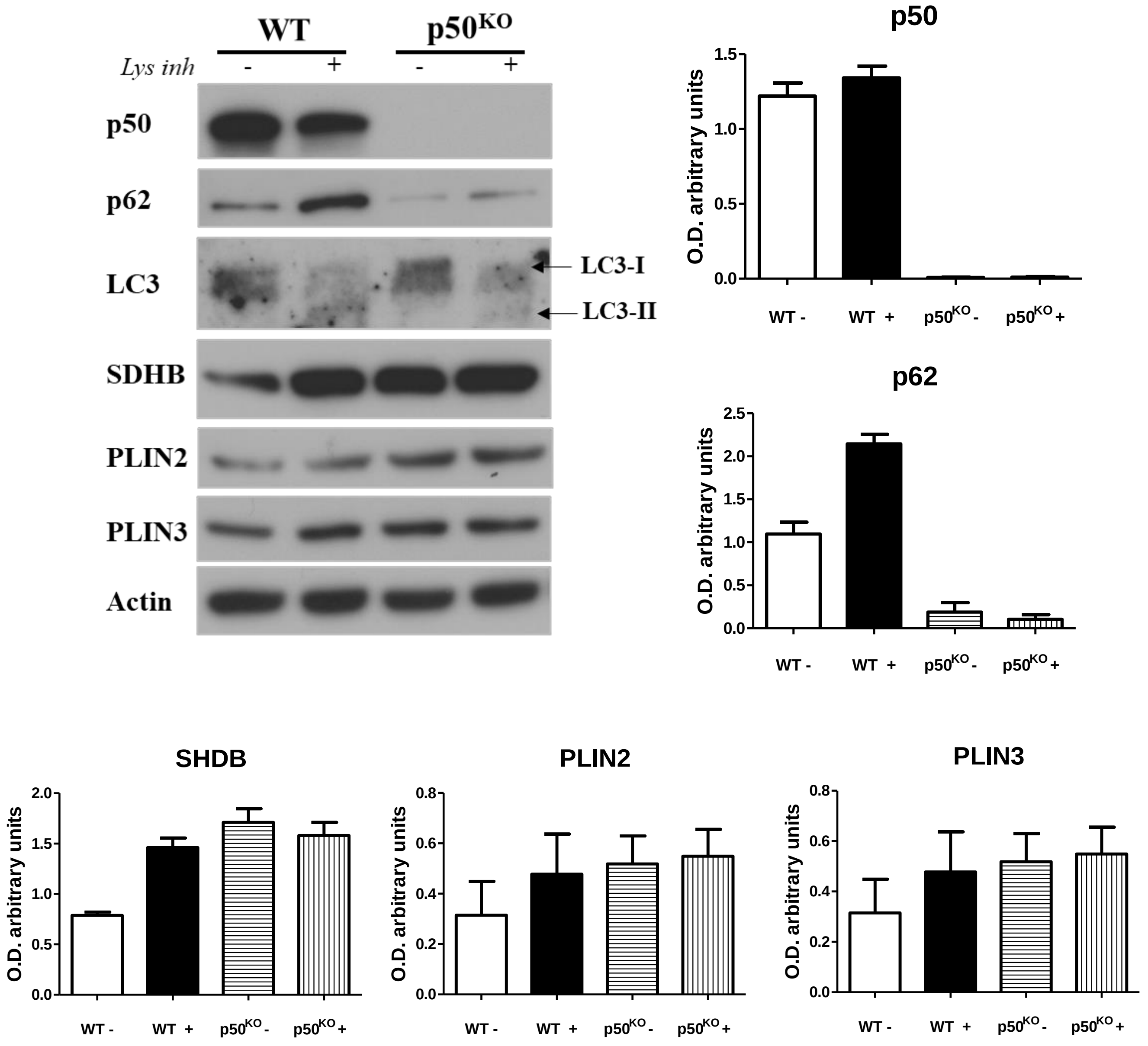
WT
p50KO
Lys inh
-
+
-
+
p50
p62
LC3
LC3-I
LC3-II
SDHB
PLIN2
PLIN3
Actin
p50
O.D. arbitrary units
1.5
1.0
0.5
0.0
WT -
WT +
p50KO -
p50KO +
p62
O.D. arbitrary units
2.5
2.0
1.5
1.0
0.5
0.0
WT -
WT +
p50KO -
p50KO +
SHDB
O.D. arbitrary units
2.0
1.5
1.0
0.5
0.0
WT -
WT +
p50KO -
p50KO +
PLIN2
O.D. arbitrary units
0.8
0.6
0.4
0.2
0.0
WT -
WT +
p50KO -
p50KO +
PLIN3
O.D. arbitrary units
0.8
0.6
0.4
0.2
0.0
WT -
WT +
p50KO -
p50KO +

FIGURE LEGENDS

Supplementary Figure 2. Original blots. All the original blots used in this manuscript are shown. The squares highlight the fragments cropped and included in the corresponding figures.

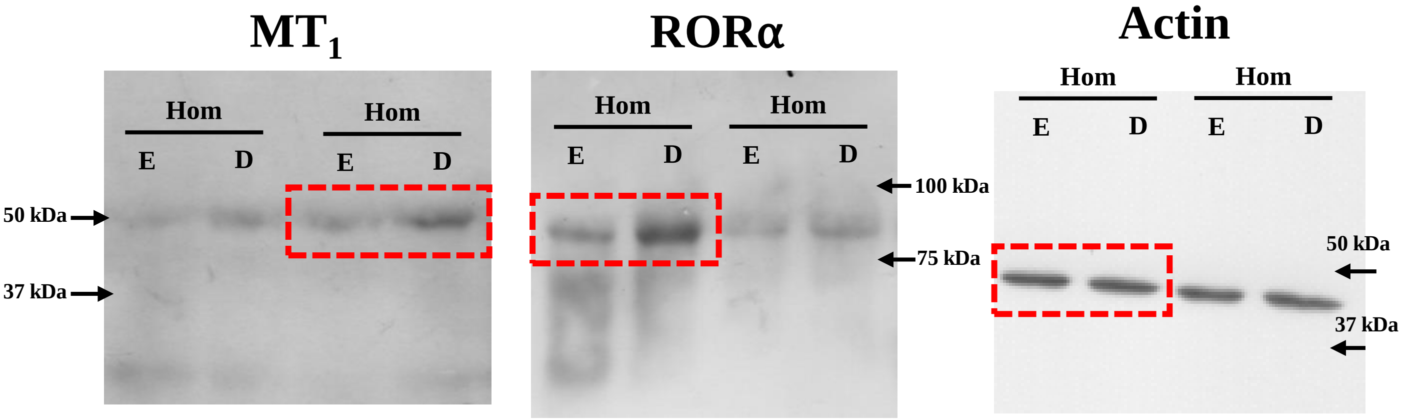

García-Macia et al. Fig. 1a

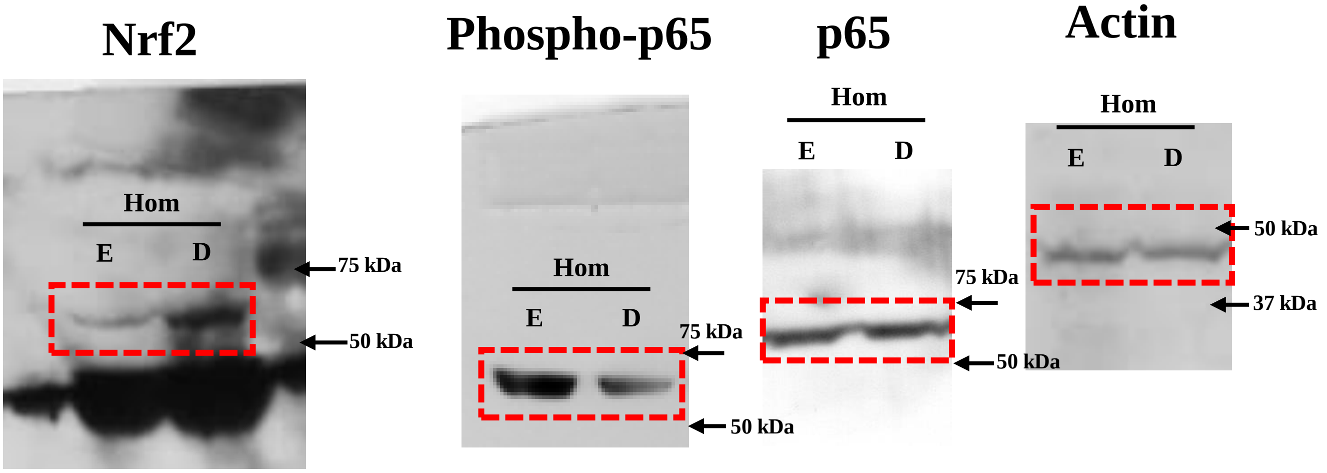

García-Macia et al. Fig. 1b

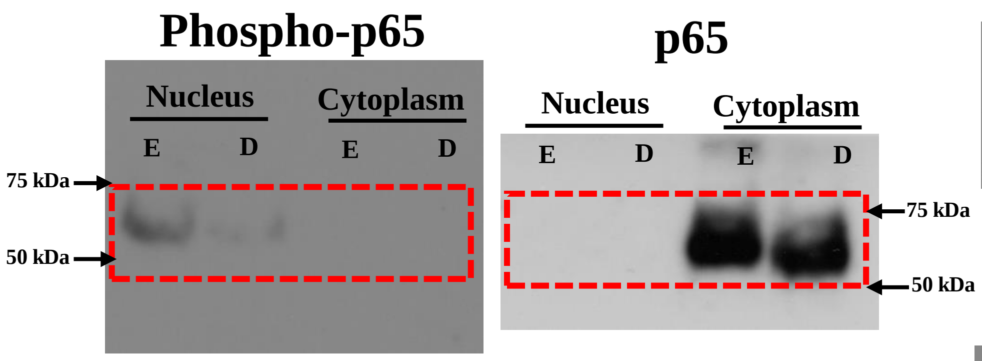

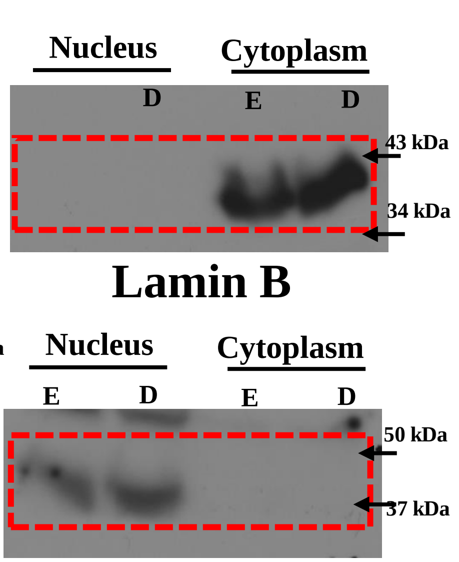

García-Macia et al. Fig. 1c

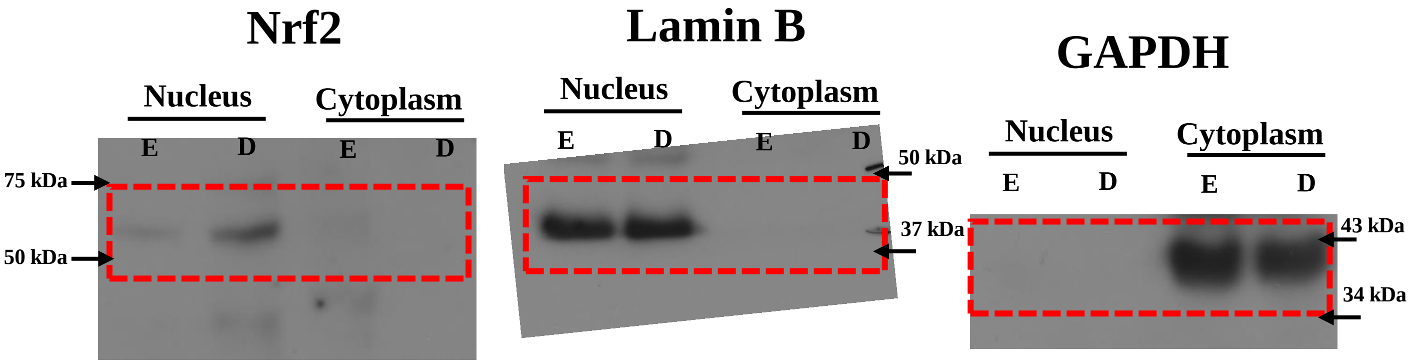

García-Macia et al. Fig. 1d

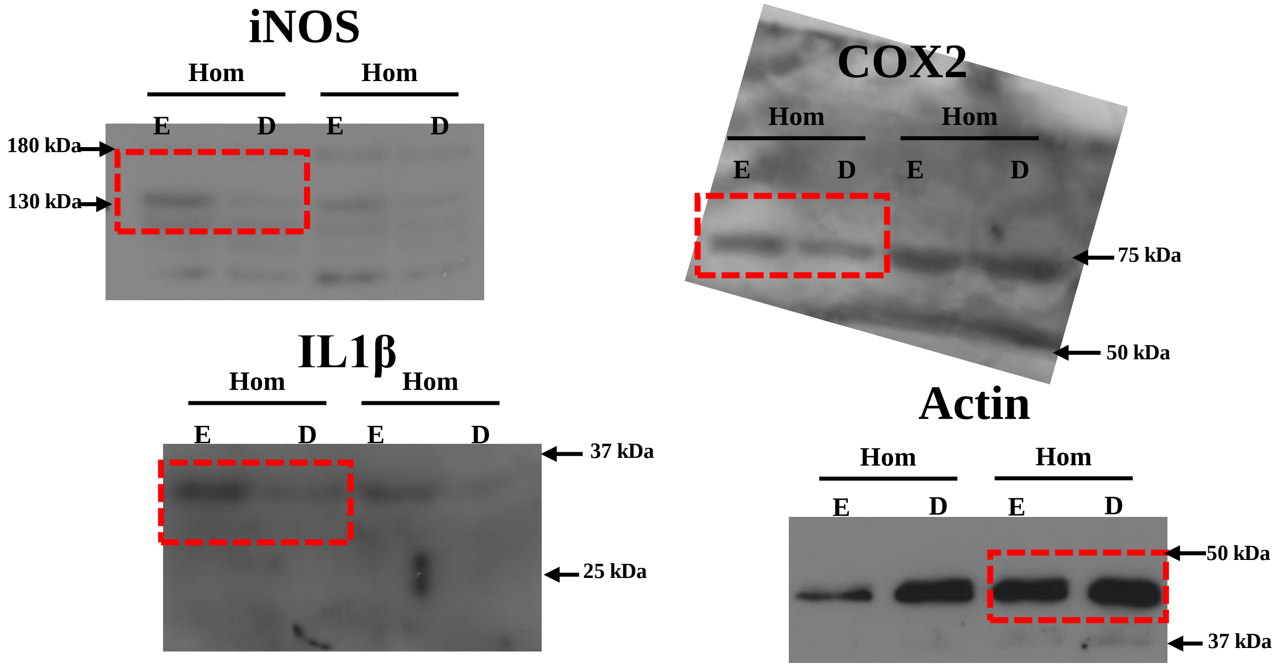

García-Macia et al.   Fig. 1e

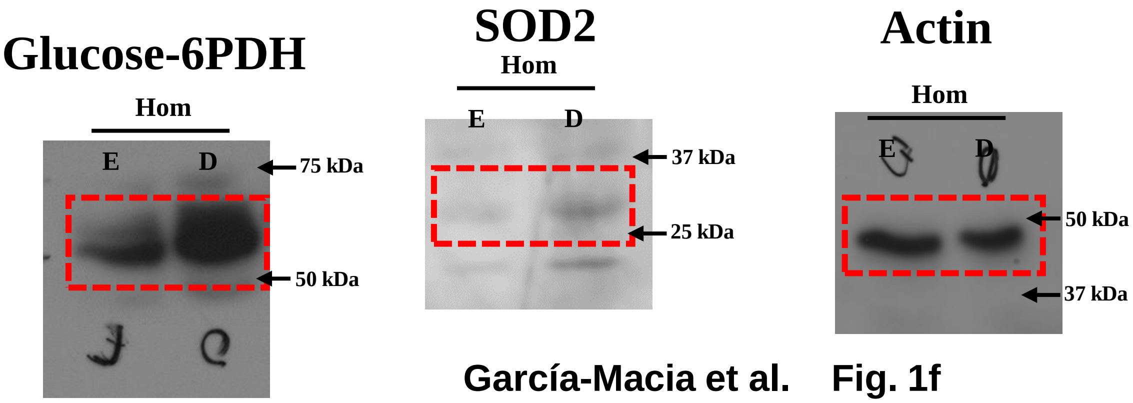

García-Macia et al.   Fig. 1f

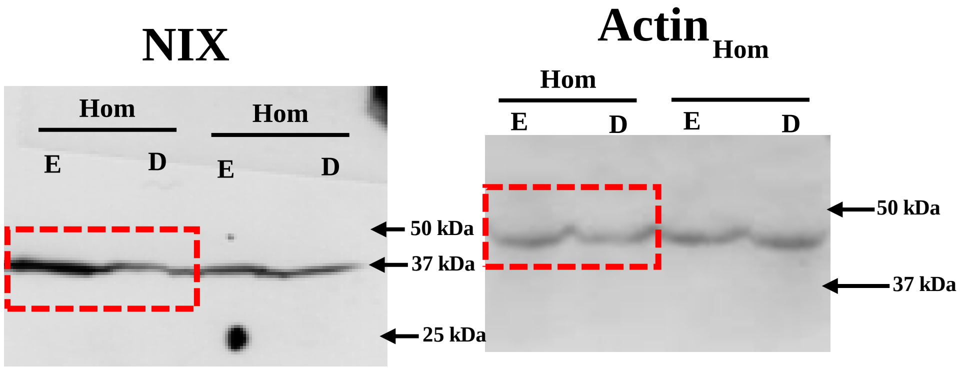

García-Macia et al.   Fig. 2c

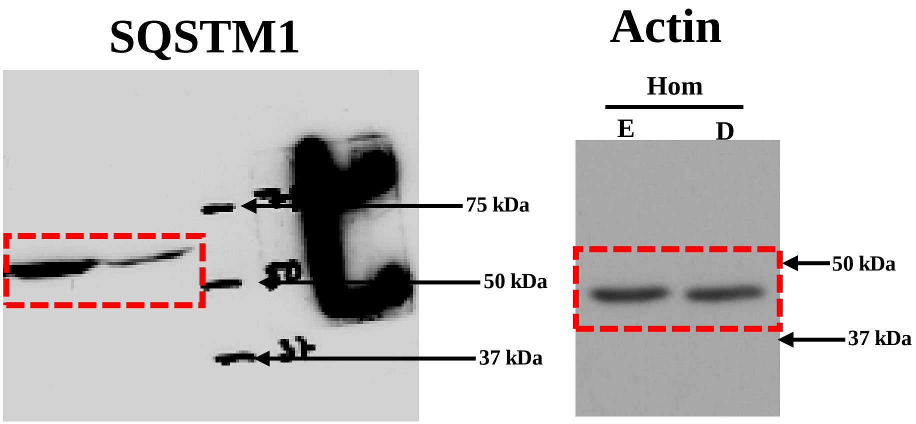

García-Macia et al.   Fig. 3a

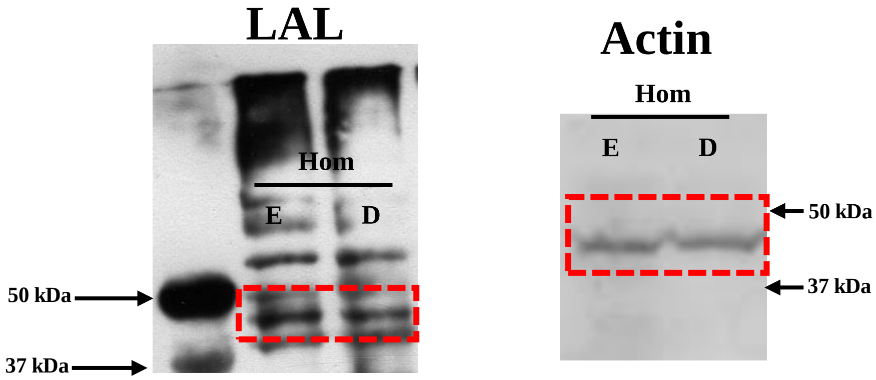

García-Macia et al. Fig. 3c

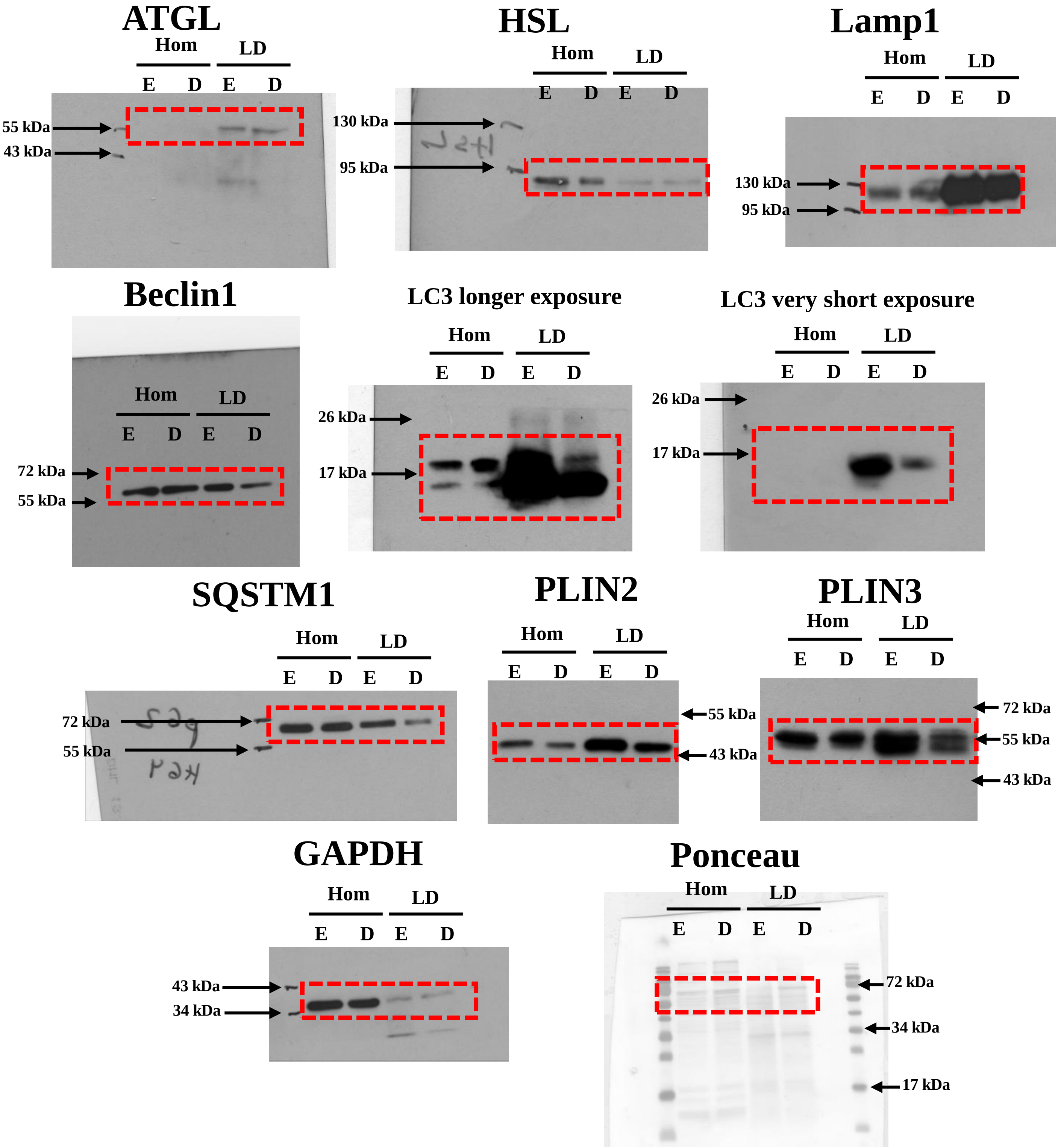

García-Macia et al. Fig. 3e

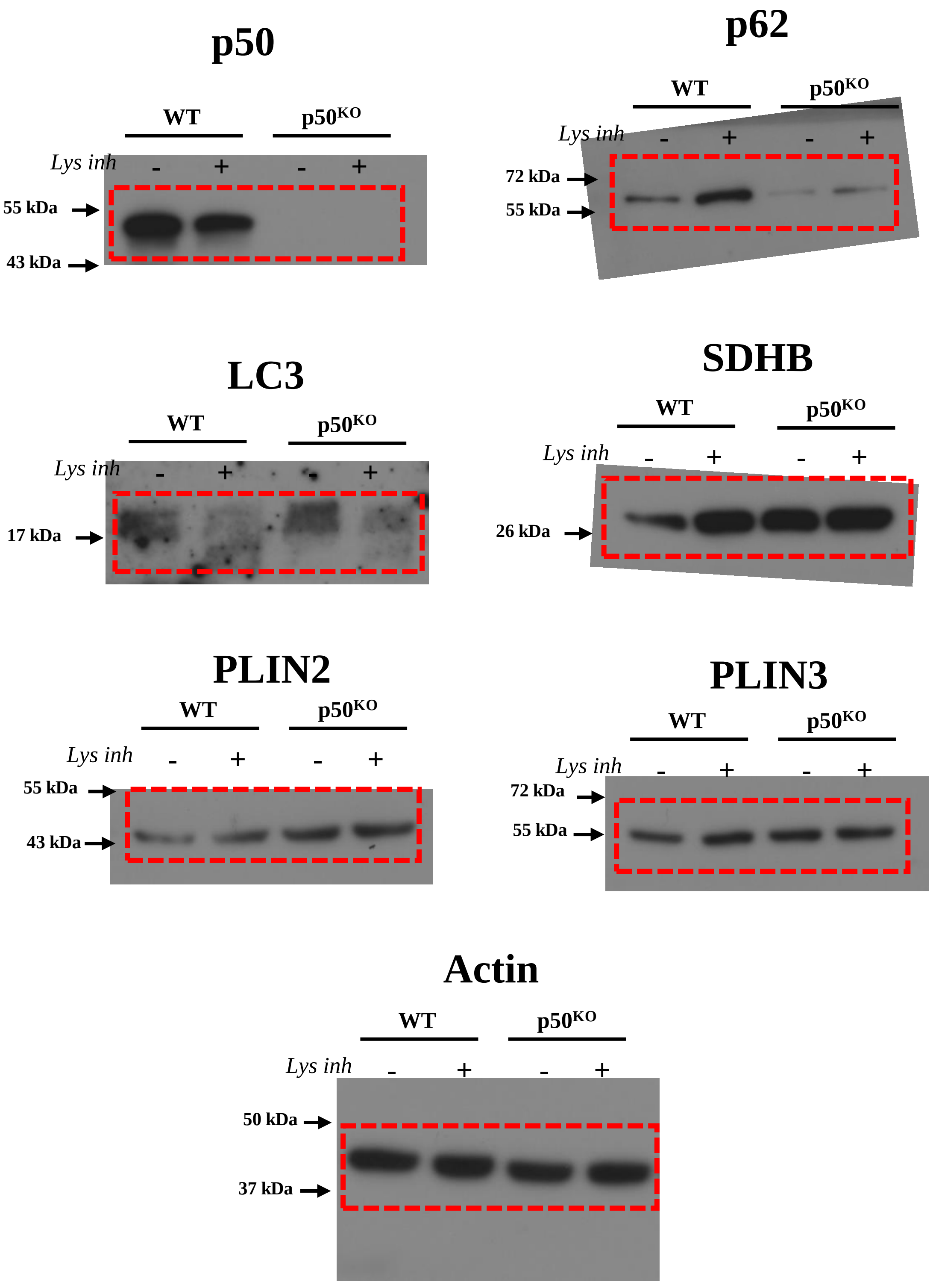

García-Macia et al. Fig. S1
